# Improved data, methods and tools for the 2007 HIV and AIDS estimates and projections

**DOI:** 10.1136/sti.2008.032573

**Published:** 2008-07-22

**Authors:** P D Ghys, N Walker, W McFarland, R Miller, G P Garnett

**Affiliations:** 1 UNAIDS, Geneva, Switzerland; 2 Johns Hopkins Bloomberg School of Public Health, Baltimore, Maryland, USA; 3 San Francisco Department of Public Health, San Francisco, California, USA; 4 Centre for Sexual Health and HIV Research, Department of Population Sciences and Primary Care, Royal Free and University College Medical School, University College London, London, UK; 5 Imperial College, London, UK

Dramatic changes in global HIV and AIDS estimates were publicised at the end of 2007,^1^ with a notable downward adjustment in the estimated number of people living with HIV (PLHIV) from 39.5 million (range 34.1–47.1 million) published in 2006^2^ to 33.2 million (range 30.6–36.1 million).^1^ Both the original and revised estimates are by any measure catastrophic; however, the new lower estimates come at a time when global disease burden estimates are under intense scrutiny.^3^ ^4^ While the *2007 AIDS Epidemic Update* report noted that the downward adjustments were the result of better data leading to changes in assumptions and thereby estimates, we recognise the need for the highest level of transparency and opportunity for scientific critique. Since 2004 we have provided detailed descriptions of the tools and assumptions used in generating HIV and AIDS estimates, as well as the data and analyses underpinning these assumptions.^5^^–^7 This current supplement assembles important new data relating to several assumptions used for the new HIV and AIDS estimates. By bringing together a new collection of methodological papers in this supplement, we aim to provide easy access to the scientific basis underlying the latest HIV and AIDS estimates for 2007.^1^ ^8^ ^9^

The process of synthesising new data, reviewing assumptions and providing guidance on methods is driven by the UNAIDS Reference Group on Estimates, Modelling and Projections.^10^ This group, which was established in 1999 and is made up of academics, researchers and public health practitioners, has led the development of several modelling and estimation packages (for example, the Workbook, EPP and the AIM module in Spectrum) that are used by countries and UNAIDS and the World Health Organization in preparing national estimates. The group meets yearly to review the major themes providing inputs to the estimation process and it is this process that has stimulated many of the papers in this supplement.

Based on the recommendations of the Reference Group, UNAIDS and WHO, along with other partners, hold workshops for national epidemiologists who are trained on the new methods and assumptions and the new versions of the modelling and estimation software. These workshops are held every two years and ensure that national epidemiologists have access to the most recent recommendations of the Reference Group and are trained in the use of the new tools. This process ensures that estimates made for different countries are comparable, as common assumptions, definitions and procedures have been used, although the accuracy of estimates across countries will vary owing to differences in data availability and the level of the epidemic.

Several papers in this supplement synthesise the data from multiple sources that have led to recent changes in assumptions behind the global HIV estimates. For example, one major adjustment is in the calibration of HIV prevalence measured at antenatal clinics (ANC) in countries with generalised epidemics, based on a comparison of HIV prevalence from ANC to national population-based surveys,^11^^–^13 which has reduced the overall estimate of the number of people living with HIV. A further change, which has lowered the historical estimate of incidence generating the current prevalence, is based on the longer survival of PLHIV.^14^^–^16 In combination with the national household-based survey in India from 2006, which prompted the country to lower its estimate of national HIV prevalence,^17^ these changes have resulted in drastic reductions in estimates of PLHIV, new HIV infections and AIDS mortality.^1^ In contrast, another important change relates to the quantification of the number of people eligible for antiretroviral therapy (ART). With the overall longer survival,^14^^–^16 and the data and analyses leading to the adoption of a longer assumed period from seroconversion to ART eligibility and from ART eligibility to death,^14^ ^18^ estimates of the number of people in need of ART have been revised and, as of the end of 2007, stand at 9.7 million (range 8.7–11 million) for low-income and middle-income countries^8^ compared to the previous estimate for 2006 of 7.1 million (6–8.4 million).^19^

The eART-linc collaboration writing group, comprising Wandel and colleagues,^18^ presents an individual participant data meta-analysis of the time from seroconversion to ART eligibility and from ART eligibility to death in the absence of ART. Unfortunately it was not possible with the available data to estimate these times for the full WHO criteria (CD4 <200 cells ×10^6^/l, or WHO stage 4, or 200< CD4 cells ×10^6^/l <350 and WHO stage 3).^20^ The time from seroconversion to ART eligibility is estimated at 66% and 48% of the total survival time for eligibility criteria CD4 <200 cells ×10^6^/l and CD4 <275 cells ×10^6^/l, respectively. This supports the implementation in Spectrum of the time from seroconversion to ART eligibility as a distribution with a median of 8 years out of 11 years total survival for most countries and 6.5 years out of 9 years for countries where HIV subtype E is dominant.^14^ These new parameter estimates for ART needs among adults are substantiated by independent analyses of a cohort in Masaka, Uganda, suggesting a 35-month period from ART eligibility to death (in the absence of ART), based on the full WHO criteria.^21^ Two published studies with a community-based assessment of the prevalence of the need for ART in adults allow for validation of the new parameter estimates. Applying the new Spectrum methods and assumptions to Malawi yields 31% (range 26%–35%) of adult PLHIV (>15 years) who are eligible for ART in 2006, while the empirical estimates for the same year in Karonga district were 38% for the Malawi national programme criteria (CD4 <250 cells ×10^6^/l or WHO stage 3/4), 34% for the full WHO criteria and 29% for CD4 <200 cells ×10^6^/l as the criterion among 18–59 year olds.^22^ Applying the new Spectrum methods and assumptions to South Africa yields 14% (range 13%–16%) and 17% (range 15%–18%) of 15–49-year-old PLHIV who were eligible for ART in 2001 and 2002, respectively, while the empirical estimate was 9.5% (95% CI 6.1% to 14.9%) among 15–49-year-olds in early 2002 in a township near Johannesberg for the CD4 <200 cells ×10^6^/l criterion only^23^—the proportion corresponding to the full WHO criteria is expected to be somewhat larger. Therefore, the new assumptions seem to provide reasonable results, and should be preferred over the previous set of assumptions.^24^

Given the past revisions in global HIV estimates and our increasing emphasis on the range of plausible estimates, we recognise the increasing need for improved and more realistic uncertainty bounds. Building on previous methods for deriving uncertainty bounds around the HIV and AIDS estimates, a new Bayesian approach that makes full use of the empirical data for each country is implemented in the latest version of the Estimation and Projection Package (EPP).^12^ ^13^ This is complemented by use of Monte Carlo methods in Spectrum for the uncertainty around HIV prevalence in concentrated epidemics, and around all other indicators for both types of epidemics.^14^

This supplement includes an illustrative example in which some of the methods described here have been applied to data from Ukraine.^25^ It should be noted that the Ukraine estimate may be on the high side, given the strong assumptions for the sexual partners of the people in the groups at higher risk, resulting in these partners representing almost a third of all people living with HIV. Also included are papers that synthesise estimates for special populations that are often missing or not distinguished in national and international estimates. For example, the global estimates resulting from the implementation of the methods described in other papers in this supplement have been combined with estimates of populations affected by emergencies in order to estimate the AIDS burden among people affected by emergencies.^26^ An example of a paper that fills a gap in the global estimates is the literature review by Caceres *et al*^27^ on the epidemiology of male same-sex behaviour in low and middle-income countries.

The discrepancies between estimates of the prevalence of maternal orphanhood between UN demographic models and demographic and health surveys (DHS) are investigated using analyses of data from Manicaland, Zimbabwe.^28^ The authors offer as a possible explanation for this discrepancy—the misreporting of foster parents as natural parents, which appears to be particularly common among foster mothers.

Two papers investigate the possible bias in HIV seroprevalence estimates from national household surveys.^29^ ^30^ Mishra *et al*, reporting from the institution that has provided technical assistance to the DHS, analysed 14 surveys and confirm their earlier finding on the basis of eight surveys that national surveys are not much affected by non-response bias.^31^ An independent analysis by Marston *et al*^30^ specifically investigated the role of mobility. Of nine national surveys examined, the bias due to non-response amounted to only 10% in the most severe case and in no case was the difference in observed versus adjusted HIV prevalence statistically significant, similar to the findings of Mishra *et al*.^29^ Still, it should be noted that the analytical approach is limited because of the lack of specific risk information about people who are eligible for participation in the survey but who were absent or did not give an interview for another reason. Mishra *et al*^29^ also investigate the potential for bias in surveys in countries with concentrated epidemics, specifically related to the exclusion of populations that are at higher risk of HIV infection from the sampling frame of the survey. They conclude that bias is unlikely to result in large differences in national prevalence—for example, in the most extreme scenario the adult HIV prevalence increased from 0.28% to 0.35% in India, and from 0.6% to 1% in Cambodia.

Two papers compare HIV prevalence estimates from ANC surveillance to population-based surveys. A first analysis was conducted for urban and rural areas for 26 countries, and concluded that adult prevalence in the surveys was approximately 0.8 of the ANC prevalence.^11^ A second analysis confirms this association, but shows that the prevalence in ANC is similar to the prevalence found in the clusters of the surveys that are near ANC, both for adults and for women.^32^ Together, these papers suggest that, besides differences in prevalence by gender, the major reason for the prevalence difference between surveys and ANC is because of the geographical non-representativeness of ANC sites included in countries’ surveillance systems, with urban ANC sites disproportionately located in the larger cities and towns, and similarly rural ANC sites leaving out the remote areas of countries that are poorly covered by antenatal services. The papers also provide the rationale for the recalibration of ANC prevalence by a factor of approximately 0.8 when making estimates of national HIV prevalence in countries without specific, local data based on the consistency of findings, as implemented in EPP.^12^ ^13^

In addition to making national estimates every two years, UNAIDS and WHO also do a systematic review of the quality of data available in countries to make their estimates of HIV and AIDS. The quality of sero-surveillance in low-income and middle-income countries is reviewed through 2007.^33^ Surprisingly, there is little evidence on a general trend in improved surveillance systems among the 127 country systems that were reviewed. Overall, 44 out of 127 countries were scored as poorly functioning systems, a number similar to that found two years earlier.^34^ However, some countries have strengthened their systems and data availability. The improvement that has occurred is primarily in countries with data from nationally representative surveys which test for HIV, mainly in sub-Saharan Africa.

Heaton *et al*^35^ present and discuss the potential and the difficulties of various modelling approaches to estimate the number of HIV infections averted by prevention programmes. This is an important question to address as governments and other organisations would like to know if the money and efforts spent on prevention have yielded tangible benefits. The authors of the paper argue that while all approaches have strengths and weaknesses, a disease-modelling approach will yield the best results and can be used to estimate infections averted.

In reviewing the estimates of HIV prevalence and the associated estimates of impact it is important to remember their function and not solely concentrate on getting the headline figure right. Key functions of the estimates are to plan for the impact of the infection and disease; to plan resource distribution to match the need for prevention, treatment and care and to understand trends in the epidemic. In revising assumptions and current estimates it is important to similarly revise historical estimates so that the trends can be explored in a consistent manner. This is built into the models used to explore the epidemiological data. However, future changes in the epidemiology of infection and biases in surveillance data brought about by increased testing and increased availability of treatment will have to be considered. Despite the large number of people currently being treated, the impact of treatment on estimates has to date been limited because it takes time for the impact of improved survival to accumulate. However, this situation is likely to change over the next two years and the methods will need to be adjusted accordingly. In addition to an increased HIV prevalence because of improved survival rather than more new infections we can expect some changes in behaviour, both risk behaviour and contact with services, brought about by high HIV testing and knowledge of serostatus.

For planning purposes we would ideally like to understand the current pattern of spread of HIV and be able to evaluate the effectiveness of prevention programmes in the short term. Unfortunately, tools to measure recent incidence beyond a limited research setting are not currently available. HIV incidence estimates, as currently calculated in a model based on HIV prevalence trends,^14^ are likely to be reasonably precise for the period up to several years ago but become much less reliable for recent and current years. While a method has been developed to derive incidence from age-specific prevalence measured in national surveys,^36^ direct measurement of incidence remains highly desirable, although there are important challenges that need to be overcome.^37^ ^38^ This extends beyond a technological problem since the required sample sizes, especially in low prevalence settings, to understand incidence would be very large.

Estimates for generalised epidemics are now much more precise than those for concentrated epidemics, where there is much uncertainty around the size of specific groups, and the representativeness of HIV sentinel surveillance among these groups. A number of methods to estimate the size of these groups are available, but they can be demanding in terms of required data and analytical capacity. There is currently no consensus on the best and most appropriate methods which means rigour is often compromised when deciding on which estimates to use. These difficulties in assessing the current state of the epidemic in low prevalence situations also undermine attempts to compare prevalence estimates over time and understand the trends in the epidemic in these locations. These problems have been less of a focus in this supplement because of the dramatic changes in estimates for generalised epidemics and their dominance in the estimates. None the less, in planning prevention, treatment and care, understanding the situation in concentrated and low-level epidemics is important and more work is required in this area.

In conclusion, we anticipate that further improved data, along with changes in HIV prevention and care, will cause future changes in both assumptions and the estimates derived from them. As we hope we have illustrated in this supplement, we are committed to further improve estimates and to continue to provide the scientific community, policy-makers and the wider public with the opportunity to carefully examine the evidence behind this work.
